# Gradient Porous PVA/CB Composites for High-Performance Flexible Piezoresistive Sensors

**DOI:** 10.3390/polym18131630

**Published:** 2026-06-30

**Authors:** Changze Mei, Tian Zhang, Yong Zhang

**Affiliations:** 1 State Key Laboratory of Advanced Glass Materials, School of Materials Science and Engineering, Wuhan University of Technology, Wuhan 430070, China; 2 Center for Smart Materials and Device Integration, Wuhan University of Technology, Wuhan 430070, China; 3 Electronic Information School, Wuhan University, Wuhan 430072, China; 4 Suzhou Institute of Wuhan University, Suzhou 215000, China

**Keywords:** pressure sensor, gradient composite, piezoresistive, sensitivity

## Abstract

Flexible piezoresistive sensors often face a trade-off between sensitivity and working range. In this work, a gradient porous poly(vinyl alcohol)/carbon black (PVA/CB) composite was fabricated via a simple sugar-templating method. The bilayer structure consists of a small-pore layer and a large-pore layer, enabling sequential deformation under external pressure. As a result, the sensor exhibits a sensitivity of −3.05 kPa^−1^ in the low-pressure range (0–20 kPa) and maintains a stable response up to 120 kPa. Compared with uniform porous structures, the gradient design shows improved performance in the medium- and high-pressure ranges. The sensor also demonstrates good repeatability, fast response, and stability over 1000 cycles. Practical applications including respiration monitoring, vocal vibration detection, and motion sensing are demonstrated. This work provides a simple and scalable approach for developing flexible pressure sensors.

## 1. Introduction

Flexible pressure sensors have become essential components in wearable electronics and human–machine interfaces [1,2,3,4,5,6,7,8]. Among various transduction mechanisms, piezoresistive sensors stand out due to their simple structure, facile signal readout, and low-cost fabrication, making them promising for health monitoring, motion detection, and electronic skin applications [9,10,11,12]. However, conventional piezoresistive sensors based on dense polymer/conductive filler composites suffer from limited compressibility, resulting in low sensitivity and early saturation under high pressures, which hinders their use in wide-range detection scenarios [13,14,15].

Introducing porous structures into the sensing layer has been widely explored to enhance sensitivity [16,17,18]. The abundant voids within porous materials enable substantial deformation under external forces, where the buckling and collapse of pore walls effectively amplify the reconstruction of conductive networks and thus the resistance change [19,20,21]. For instance, Liu et al. developed polyimide/MXene composite aerogels achieving high sensitivity to subtle pressures [22]. Wei et al. reported bioinspired carbon aerogels with excellent piezoresistive performance [23]. While porous structures significantly improve sensitivity, the uniform pore size distribution introduces a new challenge: all pores deform simultaneously under low pressure, contributing to ultrahigh sensitivity, but they rapidly densify under increasing pressure, leading to premature saturation and a narrow linear range [24,25,26]. Balancing high sensitivity with a broad working response remains a critical challenge in the field.

Inspired by gradient structures in biological tissues such as skin and bone, researchers have explored sensing materials with graded pores to achieve sequential deformation under varying pressures [27,28,29]. Shi et al. designed multiscale hierarchical structures that moderately improved the linear sensing range [27]. Chen et al. exploited nonlinear synergy effects to extend the linear response range of pressure sensors to 1.4 MPa [28]. Zhu et al. combined freezing and directional diffusion techniques to fabricate gradient hydrogels for wide-range pressure sensing [29]. These studies demonstrate that gradient architectures, where different regions of the material bear deformation successively, offer an effective route to reconcile high sensitivity and broad working range. However, current methods for constructing gradient structures often involve complex procedures or expensive equipment, limiting their practical scalability.

Herein, we report a simple and eco-friendly sugar-templating method to fabricate a bilayer gradient porous PVA/CB piezoresistive sensor. The key novelty of this work lies in the sequential deformation mechanism enabled by the gradient architecture: a small-pore bottom layer and a large-pore top layer. This design allows the sensor to achieve high sensitivity at low pressure and a broad working range at high pressure simultaneously—addressing the long-standing trade-off between sensitivity and working range in flexible piezoresistive sensors. Compared with conventional uniform porous structures, our gradient design offers a distinct advantage in maintaining detectable response under high pressure. This work provides a low-cost, scalable strategy for developing high-performance flexible pressure sensors.

## 2. Experimental Section

### 2.1. Materials and Chemicals

Poly(vinyl alcohol) (PVA, 1799 type, degree of polymerization: 1700; alcoholysis degree: 98–99%) was purchased from Aladdin Chemical Reagent Co., Ltd., Shanghai, China Carbon black (Super P, particle size 40 nm, purity > 99%) was supplied by TIMCAL Ltd., Bodio, Switzerland). Glycerol (analytical grade) and anhydrous ethanol (analytical grade, ≥99.7%) were obtained from Sinopharm Chemical Reagent Co., Ltd., Shanghai, China Borax (analytical grade) was purchased from Aladdin Chemical Reagent Co., Ltd. White granulated sugar was purchased from a local supermarket and sieved to obtain fine sugar (60–80 mesh, 180–250 μm) and coarse sugar (40–60 mesh, 250–380 μm). Deionized water was used throughout the experiments. PVA was selected as the polymer matrix for several reasons. First, its good water solubility and film-forming ability enable easy processing via the sugar-templating method. Second, its hydroxyl groups allow crosslinking with borax to form a stable hydrogel network. Third, PVA is biocompatible, non-toxic, and low cost, making it suitable for wearable applications. Compared with recently reported biopolymer-based pressure sensors, such as cellulose ionogels [30] and enzymatically crosslinked gelatin organohydrogels [31], our PVA/CB system offers better water solubility and processability via sugar-templating, which enables the construction of a bilayer gradient porous structure.

### 2.2. Preparation of Sucrose Templates

Granulated sugar was first dried at 60 °C for 2 h. The sugar crystals were then sieved using standard sieves with mesh sizes of 40, 60, and 80 meshes. The fractions retained on the 40-mesh sieve but passing through the 60-mesh sieve (particle size 250–380 μm) were collected as coarse sugar templates, while the fractions retained on the 60-mesh sieve but passing through the 80-mesh sieve (particle size 180–250 μm) were collected as fine sugar templates. To reduce their dissolution rate in aqueous PVA solutions, the sugar particles were immersed in anhydrous ethanol for 30 s, followed by filtration and air-drying.

### 2.3. Preparation of CB/PVA Slurry

PVA solution (10 wt%) was prepared by dissolving 10 g of PVA in 90 g of deionized water at 95 °C under mechanical stirring for 3 h until complete dissolution. The solution was then cooled to 40 °C. Glycerol (20 wt% relative to PVA solid) was added and stirred for 5 min. Carbon black (15 wt% relative to PVA solid) was then slowly added under stirring. The CB concentration of 15 wt% (relative to PVA solid) was chosen based on preliminary experiments. CB contents below 10 wt% resulted in excessively high initial resistance (>1 MΩ), which compromised reliable piezoresistive signal acquisition. Conversely, CB contents above 20 wt% led to aggregation of CB particles, poor dispersion, and reduced mechanical flexibility. The 15 wt% formulation balanced electrical conductivity and mechanical integrity, and was therefore adopted for this study. Then, 20 min of magnetic stirring and 30 min of ice-bath ultrasonication were used to achieve uniform dispersion. The sugar templates (mass ratio of sugar to PVA solid = 1:1) were added and mixed thoroughly. Finally, borax solution (1 wt% in water, 0.8 wt% relative to PVA solid) was added as a crosslinker with rapid stirring for 30 s.

### 2.4. Fabrication of Gradient Porous CB/PVA Films

The gradient bilayer porous film was fabricated using a sequential casting process. First, approximately 10 mL of the PVA/CB composite slurry was mixed with an appropriate amount of the pretreated fine sugar templates (60–80 mesh, 180–250 μm). Borax solution (1 wt%) was added at 0.8 wt% relative to PVA, rapidly stirred for 30 s, and immediately cast into a polytetrafluoroethylene (PTFE) mold. The mold was placed in an oven at 40 °C for about 3 h until the surface became semi-dry. Subsequently, the remaining 10 mL of the PVA/CB slurry was mixed with an appropriate amount of the pretreated coarse sugar templates (40–60 mesh, 250–380 μm). After adding the same proportion of borax solution and stirring for 30 s, the mixture was immediately cast onto the semi-dry bottom layer and leveled with a glass plate. The mold was then placed back in the 40 °C oven for 24 h for complete curing. After demolding, the sample was immersed in 60 °C deionized water, with water changed every 20 min for a total of 5 times, to thoroughly dissolve the sugar templates. The sample was then sequentially immersed in 50%, 75%, and 100% ethanol for 30 min each, followed by air-drying at room temperature for 12 h. The final product was denoted as GPM. For comparison, two types of uniform porous films were prepared using the same procedure: one using only coarse sugar templates (denoted as UPM-Coarse) and the other using only fine sugar templates (denoted as UPM-Fine).

### 2.5. Characterization and Electrical Measurement

The morphology of the porous films was observed using scanning electron microscopy (SEM, JSM-7610F Plus, JEOL Ltd., Akishima, Tokyo, Japan) at an accelerating voltage of 5 kV after sputter-coating with gold. Mechanical properties were tested using a universal testing machine (MST Criterion 44, MTS Systems Corporation, Eden Prairie, MN, USA) at a loading rate of 2 mm/min. For tensile tests, samples were cut into rectangular strips. For compression tests, samples were cut into 10 mm × 10 mm squares with a thickness of approximately 4 mm. Pressure sensing performance was evaluated using a custom-built setup consisting of a uniaxial motion stage, a digital force gauge (YKL-20N, Shanghai, China), and a digital multimeter (DMM6500, Keithley Instruments, Solon, OH, USA). Pressure was calculated as force divided by the sensor area (P = F/A). The relative resistance change was defined as ΔR/R_0_ = (R − R_0_)/R_0_, where R_0_ is the initial resistance without pressure. Sensitivity was calculated as S = (ΔR/R_0_)/ΔP in different pressure ranges, where ΔR/R_0_ is expressed in percentage (not as a decimal). Response and recovery times were defined as the time required for the resistance change to reach 90% of the steady-state value upon loading and to recover to 10% above the initial value upon unloading, respectively. Cyclic stability was tested by applying a pressure of 50 kPa at a frequency of 0.5 Hz for 1000 cycles.

### 2.6. Application Demonstration

For respiration monitoring, the sensor was attached to the inner side of a mask or below the nasal cavity. For vocal vibration detection, the sensor was attached to the thyroid cartilage position of the throat. For joint motion detection, the sensor was attached to a finger joint, and resistance changes were recorded during finger bending at different angles (0°, 30°, 60°, and 90°). All resistance signals were recorded using a digital multimeter (DMM6500, Keithley, USA) connected to a computer.

### 2.7. Fabrication and Structure

**Figure 1:**
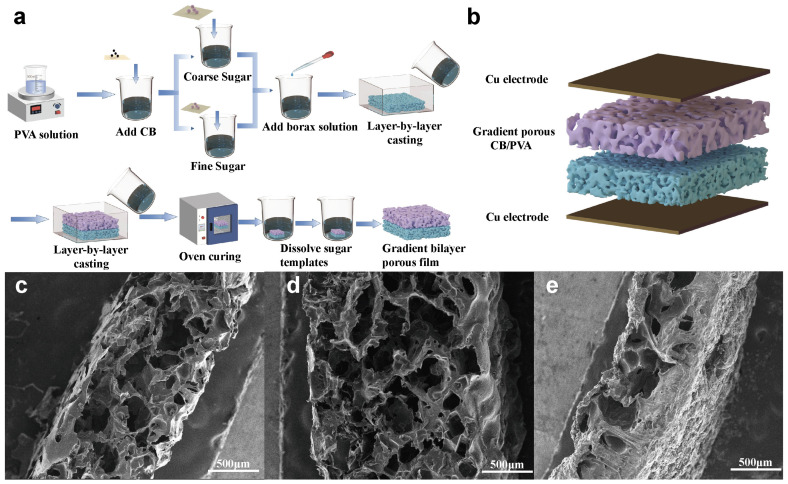
(**a**) Schematic illustration of the fabrication process of the gradient bilayer porous PVA/CB film via sequential sugar templating. (**b**) Schematic diagram of the device structure with copper electrodes and PET encapsulation. (**c**) Cross-sectional SEM image of the gradient porous membrane. (**d**) Cross-sectional SEM image of the uniform porous membrane. (**e**) Surface SEM image of the gradient porous membrane.

Figure 1a illustrates the fabrication procedure for the gradient bilayer porous PVA/CB film. Initially, a 10 wt% PVA aqueous solution was prepared at 95 °C under continuous stirring. Upon cooling to 40 °C, glycerol (20 wt% relative to PVA) and carbon black (15 wt% relative to PVA) were sequentially dispersed into the mixture to impart flexibility and conductivity. To construct the gradient architecture, this suspension was divided into two portions, which were respectively mixed with fine (60–80 mesh, 180–250 μm) and coarse (40–60 mesh, 250–380 μm) sugar templates. Following the addition of a borax crosslinker to stabilize the polymer network, the film was assembled via a two-step casting method: the fine-template mixture was poured into a mold and allowed to partially set, creating a tacky surface onto which the coarse-template mixture was then cast. Once fully cured, the sugar particles were leached out using deionized water [26]. The resulting bilayer porous film was finally subjected to solvent exchange with ethanol and dried at room temperature.

As schematically depicted in Figure 1b, the device features a typical sandwich architecture, where the gradient porous film is positioned between copper electrodes and encapsulated by PET layers. Structurally, the active layer exhibits a distinct pore-size gradient from the bottom (180–250 μm) to the top (250–380 μm). This hierarchical design dictates a sequential deformation mechanism under applied stress: the larger pores in the top layer, possessing thinner pore walls, deform readily at low pressure to deliver high initial sensitivity, whereas the smaller pores in the bottom layer gradually compress at elevated pressures, thereby substantially broadening the working range [16,28]. The cross-sectional morphologies of the GPM and the uniform membrane are shown in Figure 1c and Figure 1d, respectively, while the surface morphology is presented in Figure 1e. ImageJ 1.54g analysis of cross-sectional SEM images reveals that the bottom (small-pore) layer has an average pore diameter of 240 ± 28 μm, while the top (large-pore) layer has an average pore diameter of 355 ± 42 μm (50 pores per layer). The thickness of the top (large-pore) layer is 560 ± 22 μm, and the thickness of the bottom (small-pore) layer is 460 ± 32 μm, measured from three different positions in the cross-sectional SEM images. The porosity of the GPM is approximately 68% (Figure S1).

## 3. Results and Discussion

### 3.1. Mechanical and Electrical Properties

**Figure 2:**
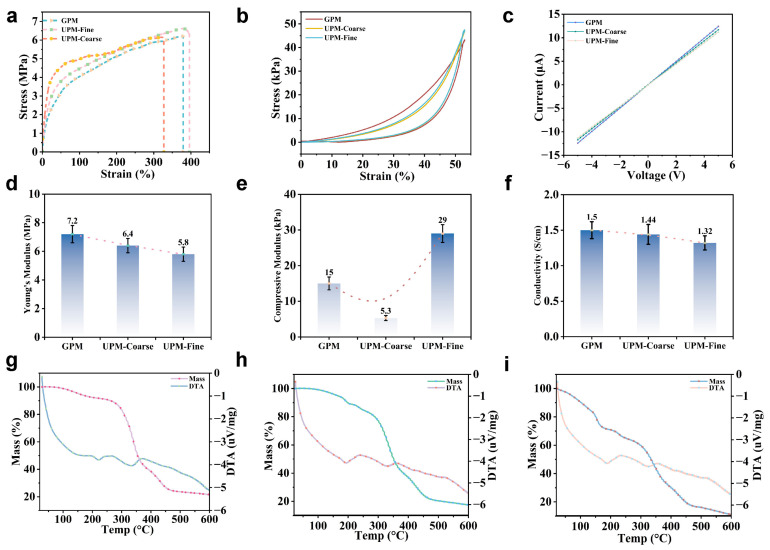
(**a**) Tensile stress–strain curves of UPM-Fine, UPM-Coarse, and GPM. (**b**) Compressive stress–strain curves of UPM-Fine, UPM-Coarse, and GPM. (**c**) I-V characteristic curves of UPM-Fine, UPM-Coarse, and GPM. (**d**) Young’s modulus of UPM-Fine, UPM-Coarse, and GPM. (**e**) Compressive modulus of UPM-Fine, UPM-Coarse, and GPM. (**f**) Electrical conductivity of UPM-Fine, UPM-Coarse, and GPM. (**g**–**i**) TGA curves of (**g**) UPM-Coarse, (**h**) UPM-Fine, and (**i**) GPM.

The mechanical behavior of the three membranes shows a clear structure-dependent trend (Figure 2a). The UPM-Fine exhibits the highest fracture elongation of 395%, which can be attributed to its thinner pore walls that facilitate polymer chain orientation during stretching. The GPM follows with 380%, while the UPM-Coarse shows the lowest value of 327%. Interestingly, the GPM possesses the highest Young’s modulus (7.2 MPa), compared to 6.4 MPa for the UPM-Coarse and 5.8 MPa for the UPM-Fine. This suggests that the gradient structure does not simply behave as an intermediate between the two uniform membranes; instead, the bilayer configuration creates a synergistic stiffening effect. The tensile strengths of all three membranes are comparable (6.1–6.5 MPa), indicating that the carbon black reinforcement plays a dominant role, with pore structure having only a limited influence. All mechanical and electrical tests were performed on at least three independent samples for each membrane type (UPM-Fine, GPM, and UPM-Coarse; n = 3). The data in Figure 2d–f are presented as mean ± standard deviation (SD).

Compression tests (Figure 2b) reveal significant differences in compressive modulus among the three membranes. The fine porous membrane (UPM-Fine) exhibits the highest compressive modulus of 29 kPa, attributed to its dense pore structure and thicker pore walls. In contrast, the coarse porous membrane (UPM-Coarse) shows the lowest value of 5.3 kPa due to its larger pore size and higher porosity. The gradient membrane (GPM) displays an intermediate compressive modulus of 15 kPa, which is approximately three times that of the UPM-Coarse but only half that of the UPM-Fine. This trend indicates that the gradient structure exhibits an intermediate compressive modulus between those of the UPM-Fine and UPM-Coarse, suggesting that the bilayer architecture combines the stiffness of the fine-pore layer with the deformability of the coarse-pore layer. The stress–strain curve of the GPM exhibits three characteristic stages typical of cellular solids: a linear elastic region (0–15% strain), a plateau region (15–45% strain), and a densification region (>45% strain). This behavior corroborates the sequential deformation mechanism—the large pores in the top layer deform first under low pressure, followed by the small pores in the bottom layer under higher pressure.

Electrically, all three membranes exhibit highly linear I-V curves passing through the origin (Figure 2c), confirming stable Ohmic contact between the electrodes and the sensing layer without any rectifying behavior. The GPM shows the highest conductivity of 1.5 × 10^−1^ S/cm, slightly higher than that of the UPM-Coarse (1.4 × 10^−1^ S/cm) and the UPM-Fine (1.3 × 10^−1^ S/cm) (Figure 2f). This modest increase may be attributed to the bilayer architecture, which provides additional parallel conductive pathways through the interconnected pore walls.

Thermogravimetric analysis (Figure 2g–i) reveals that all three membranes share nearly identical thermal degradation profiles. The main weight loss occurs between 300 and 400 °C, corresponding to the decomposition of the PVA backbone. At 600 °C, all samples retain approximately 21 wt%, slightly higher than the theoretical carbon black content of 15 wt%. This discrepancy is attributed to the carbonization of glycerol and the decomposition of borax. The near-overlapping nature of the three TGA curves confirms that neither the porous structure nor the gradient design compromises the thermal stability of the material—an important consideration for practical applications.

### 3.2. Piezoresistive Performance

**Figure 3:**
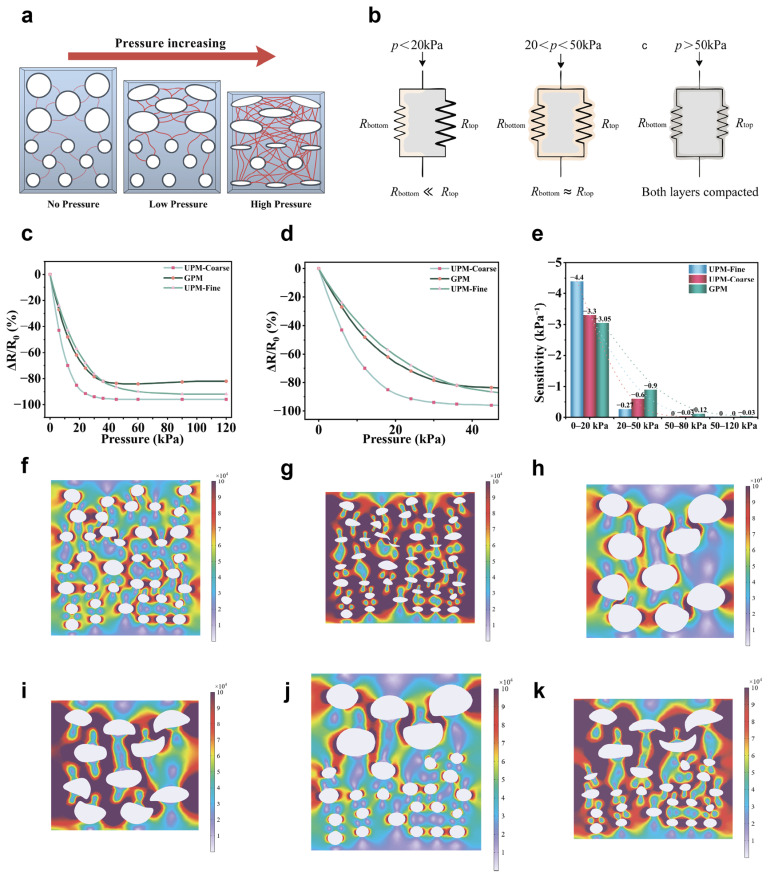
(**a**) Schematic illustration of the piezoresistive mechanism of the gradient membrane. (**b**) Equivalent parallel circuit model of the GPM. (**c**) Piezoresistive responses of GPM, UPM-Coarse, and UPM-Fine over 0–120 kPa. (**d**) Enlarged view of the 0–50 kPa region from (**c**). (**e**) Sensitivity comparison of GPM, UPM-Fine, and UPM-Coarse in three pressure ranges (0–20 kPa, 20–50 kPa, and 50–80 kPa). (**f**–**k**) Finite element simulations of stress distribution: (**f**) UPM-Fine under low stress, (**g**) UPM-Fine under high stress, (**h**) UPM-Coarse under low stress, (**i**) UPM-Coarse under high stress, (**j**) GPM under low stress, (**k**) GPM under high stress.

The piezoresistive mechanism of the gradient membrane relies on a pressure-driven, sequential deformation process (Figure 3a). In the low-pressure regime, the large-pore top layer, possessing thinner pore walls, undergoes preferential elastic buckling. This localized compression forces adjacent carbon black (CB) particles into contact, rapidly establishing new conductive pathways and triggering a sharp drop in macroscopic resistance. As the applied load transitions to the medium range, the deformation burden gradually shifts to the small-pore bottom layer. This delayed structural compression sustains the continuous reconstruction of the conductive network at a moderated rate, until both layers eventually reach full densification under high pressure, leading to signal saturation.

To quantitatively capture this sequential behavior, an equivalent parallel circuit model is proposed (Figure 3b). The total resistance $R_{\mathrm{total}}$ of the gradient architecture under applied pressure P is dictated by the parallel combination of the bottom ($R_{\mathrm{bottom}}$) and top ($R_{\mathrm{top}}$) layers:

$$\frac{1}{R_{total}\left(P\right)}=\frac{1}{R_{bottom}\left(P\right)}+\frac{1}{R_{top}\left(P\right)}$$

Based on classical contact theory, the resistance of each layer scales inversely with the pressure-induced conductive contacts:

$$R\left(P\right)=R_{m}+\frac{R_{c}}{n\left(P\right)}$$

where $R_{m}$ represents the intrinsic resistance of the PVA matrix, $R_{c}$ is the single-junction contact resistance, and n(P) denotes the dynamically increasing number of effective conductive contacts.

Because the large-pore top layer deforms readily at low pressures, its n(P) surges abruptly, driving the high-sensitivity initial response where $R_{\mathrm{total}}\approx R_{\mathrm{top}}$ (P<20 kPa). Conversely, the small-pore bottom layer requires higher activation stresses. As the top layer saturates (20<P<50 kPa), the bottom layer gradually engages, and their parallel contribution ($R_{\mathrm{bottom}}\approx R_{\mathrm{top}}$) crucially extends the working range before ultimate densification occurs (P>50 kPa).

Experimental measurements over a 0–120 kPa sweep perfectly validate this theoretical framework. For the GPM (Figure 3c), the relative resistance change ($\Delta R/R_{0}$) plots a distinct three-stage profile: a steep plunge from 0–20 kPa, a steady decline from 20–50 kPa, and a gradual saturation toward 120 kPa (∼−90%). By contrast, while the uniform porous membrane (UPM) exhibits a marginally higher initial drop (∼−60% at 10 kPa), its structural homogeneity causes premature pore collapse (Figure 3d). Consequently, the UPM response flatlines beyond 50 kPa, capping at merely −75% at 120 kPa.

This architectural advantage becomes evident when evaluating the step-wise sensitivity (Figure 3e). In the 0–20 kPa low-pressure region, the fine porous membrane (UPM-Fine) exhibits the highest sensitivity of −4.40 kPa^−1^, followed by the coarse porous membrane (UPM-Coarse) at −3.30 kPa^−1^, and the gradient membrane (GPM) at −3.05 kPa^−1^. This trend is expected, as the thinner pore walls of the fine membrane facilitate earlier buckling under low stress.

However, the gradient design truly excels at elevated pressures. In the 20–50 kPa region, the GPM sustains a sensitivity of −0.90 kPa^−1^, significantly higher than the UPM-Coarse (−0.60 kPa^−1^) and the UPM-Fine (−0.20 kPa^−1^). The fine membrane nearly saturates in this range due to its rapidly compacted pore structure. In the 50–80 kPa region, the GPM still maintains a sensitivity of −0.12 kPa^−1^, while the UPM-Coarse drops to nearly zero (−0.03 kPa^−1^) and the UPM-Fine completely saturates (0 kPa^−1^). Over the extended 50–120 kPa range, both uniform membranes show no further response, whereas the GPM retains a small but measurable sensitivity of −0.03 kPa^−1^.

This “low-pressure sacrifice, high-pressure compensation” characteristic directly supports the equivalent circuit model: the large-pore top layer provides progressive structural engagement that delays overall densification. The fine membrane offers the highest initial sensitivity but suffers from the narrowest working range, while the gradient membrane achieves the best balance between sensitivity and working range.

To further elucidate the mechanical behavior of the three porous structures under compression, finite element analysis (FEA) was performed using COMSOL Multiphysics 6.0 Multiphysics. The PVA/CB composite was modeled as a linear elastic material with a Young’s modulus of 6.2 MPa and a Poisson’s ratio of 0.35. The bottom boundary was fixed, and a uniform compressive stress was applied to the top boundary. To simulate different pressure regimes, applied stresses of 40 kPa (representative of low stress) and 90 kPa (representative of high stress) were used. The model was discretized using tetrahedral elements with a minimum element size of 5 μm, resulting in approximately 35,000 elements. A stationary study was conducted to obtain the stress distribution contours. Figure 3f–k show the stress distribution contours of the fine porous membrane (FPM), coarse porous membrane (CPM), and gradient porous membrane (GPM) under low and high compressive stresses.

Under low stress (Figure 3f,h,j), the FPM exhibits highly concentrated stress at the pore walls with localized high-strain regions, indicating that its thin pore walls deform readily but are prone to rapid collapse. The CPM shows a more diffuse stress distribution due to its larger pore size, with stress primarily localized around the pore edges. In contrast, the GPM demonstrates a distinct gradient stress distribution pattern: stress is predominantly borne by the large pores in the top layer, while the bottom layer with small pores remains relatively undeformed. This confirms that the top large pores act as a stress buffer, delaying the deformation of the bottom layer.

Under high stress (Figure 3g,i,k), all three membranes exhibit increased stress throughout the structure. However, the FPM (Figure 3g) shows severe stress concentration leading to local collapse, consistent with its early saturation behavior in the piezoresistive response. The CPM (Figure 3i) exhibits relatively uniform stress distribution but still shows localized hotspots at pore edges. Notably, the GPM (Figure 3k) maintains a more uniform stress distribution compared to the other two membranes, with no severe local stress concentration. The top large pores are largely compacted, while the bottom small pores provide additional structural support, effectively delaying overall densification.

These simulation results directly support the experimental observations: the gradient design not only provides a sequential deformation mechanism but also enables more uniform stress distribution under high pressure, which contributes to the extended working range and enhanced sensitivity retention in the high-pressure region.

### 3.3. Dynamic Response and Cycling Stability

**Figure 4:**
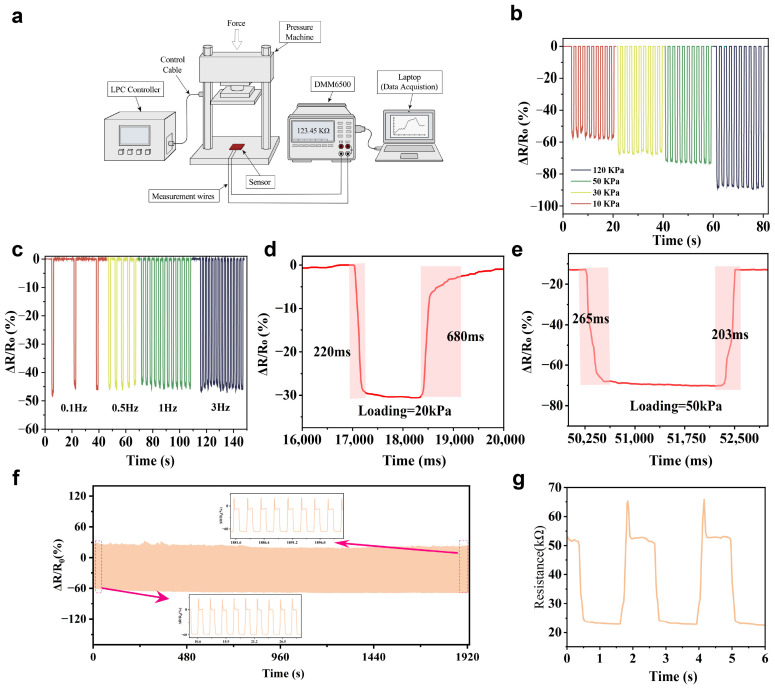
(**a**) Schematic illustration of the testing system for piezoresistive performance. (**b**) Cyclic response of the GPM under different pressures (10, 30, 50, and 120 kPa, five cycles each). (**c**) Dynamic response of the GPM under different frequencies (0.1, 0.5, 1, and 3 Hz). (**d**) Response and recovery times of the uniform porous membrane (UPM) at 50 kPa (220 ms and 680 ms). (**e**) Response and recovery times of the gradient membrane (GPM) at 50 kPa (265 ms and 203 ms). (**f**) Cycling stability of the gradient membrane over 1000 cycles at 50 kPa. (**g**) Resistance overshoot phenomenon of the gradient membrane during unloading.

The dynamic piezoresistive performance was evaluated using a customized electromechanical setup (Figure 4a), where a uniaxial motion stage applied precise compression while real-time resistance variations were continuously acquired.

The gradient porous membrane (GPM) demonstrated exceptional signal fidelity under stepped cyclic loading (Figure 4b). When subjected to varying pressures (10, 30, 50, and 120 kPa), the relative resistance change (ΔR/R_0_) amplitudes scaled reliably—from approximately −60% at 10 kPa, to −65% at 30 kPa, −72% at 50 kPa, and reaching −90% at 120 kPa. Crucially, the waveforms at each pressure plateau remained highly uniform and perfectly synchronized with the mechanical input, indicating robust pressure discrimination. The repeatability was verified by an additional independent measurement on a second GPM sensor, which yielded nearly identical results under all tested pressures (10, 30, 50, and 120 kPa).

Furthermore, the GPM exhibited remarkable frequency independence. Under a constant 10 kPa cyclic load across frequencies ranging from 0.1 to 3.0 Hz (Figure 4c), the response amplitude held steady at approximately −50% without any discernible waveform distortion or phase lag. This dynamic adaptability stems from the synergistic deformation of the hierarchical design: the coordinated interplay between the bottom small pores and top large pores ensures the complete reconstruction and dissociation of the conductive network, regardless of the loading rate.

Transient response characteristics further highlight the kinetic advantages of the bilayer architecture (Figure 4d,e). Upon a 50 kPa step load, the GPM registered a response time of 265 ms—marginally slower than the uniform porous membrane (UPM, 220 ms). However, the GPM drastically outperformed the UPM during the unloading phase, achieving a rapid recovery time of just 203 ms compared to the UPM’s sluggish 680 ms. This 70% acceleration in recovery is driven by the robust elastic rebound of the thick-walled large pores in the top layer, which rapidly drives the spatial restoration of the original conductive network.

Long-term mechanical durability, a prerequisite for practical wearable applications, was verified over 1000 continuous loading–unloading cycles at 50 kPa (Figure 4f). The GPM maintained a stable ΔR/R_0_ amplitude of approximately −72% with negligible baseline drift or signal attenuation. The nearly overlapping profiles of the initial and final 50 cycles (inset of Figure 4f) indicate the good structural stability of the gradient matrix.

During dynamic testing, an intriguing resistance overshoot phenomenon was consistently captured during the rapid unloading phase (Figure 4g). Upon sudden pressure release from 50 kPa, the resistance momentarily spiked to a value drastically higher than its initial baseline before gradually equilibrating. Specifically for the GPM, the resistance surged from its compressed state (~22 kΩ) to an overshoot peak of ~65 kΩ—an increase of nearly 200%—before returning to the ~52 kΩ baseline within 100 ms.

This overshoot behavior is an intrinsic dynamic characteristic of conductive porous elastomers. Wang et al. investigated the piezoresistive response of carbon nanotube/silicone rubber composites under multi-cycle loading and unloading, attributing the resistance overshoot to the reorganization of the contributive tunneling film network during stress release [32]. More recently, Zhang et al. developed an analytical model integrating elastic mechanics with electrical tunneling effects, demonstrating that multimodal buckling of pore walls, pore closure, microcracks, and spatial mismatch within the elastomer under large deformation collectively contribute to resistance overshooting. Their model, achieving 99.5% accuracy under 75% compression, also revealed that overshoot magnitude is highly sensitive to unloading speed and ambient temperature [33].

In the context of our gradient bilayer architecture, we ascribe this transient overshoot to the asynchronous elastic recovery of the hierarchical pores. During compression, both layers store substantial elastic strain energy. Upon abrupt unloading, the thin-walled small pores in the bottom layer rebound instantaneously, whereas the thicker-walled large pores in the top layer exhibit structural hysteresis. This temporal mismatch causes a temporary over-extension of specific pore walls beyond their stress-free positions, momentarily pulling apart the carbon black particles and creating a conductive network sparser than the virgin state. As the top layer gradually relaxes into equilibrium, the network stabilizes, and the resistance returns to baseline.

The magnitude of this overshoot exhibits a strong positive correlation with the unloading velocity. Rather than a defect, this rate-dependent characteristic unlocks potential for dual-parameter sensing. It empowers the sensor to simultaneously decode the applied pressure magnitude (via the steady-state ΔR/R_0_) and the impact/release speed (via the transient overshoot amplitude). Such bifunctionality holds immense promise for advanced human–machine interfaces and sports biomechanics, where differentiating between slow compression and ballistic impact is critical.

### 3.4. Application Demonstrations

**Figure 5:**
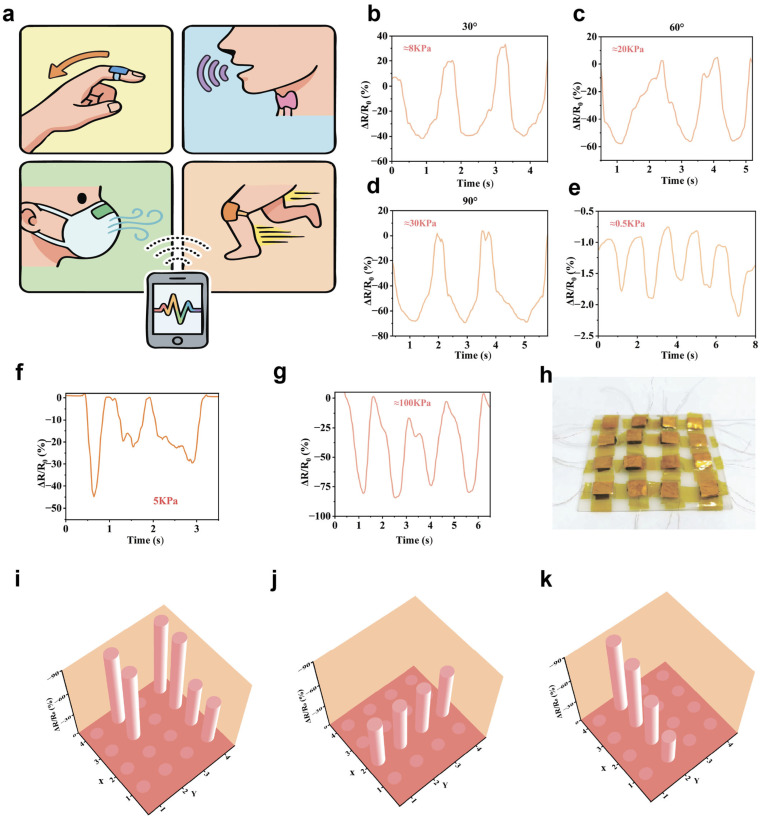
(**a**) Schematic illustration of sensor attachment positions on the human body. (**b**–**d**) Finger bending detection at different angles (0°, 30°, 60°, 90°). (**e**) Respiration monitoring. (**f**) Vocal vibration detection (monosyllabic, disyllabic, and trisyllabic). (**g**) Motion detection during running. (**h**) Schematic diagram of the 4 × 4 sensor array. (**i**) Pressure mapping under random pressure distribution. (**j**) Pressure mapping under uniform pressure distribution (horizontal direction). (**k**) Pressure mapping under gradient pressure distribution (vertical direction).

To validate the practical versatility of the gradient bilayer piezoresistive sensor, it was seamlessly integrated into various anatomical locations (Figure 5a) to monitor a broad spectrum of physiological signals and biomechanical motions. For macroscopic motion tracking, the sensor was mounted on the index finger to monitor stepwise bending angles (0°, 30°, 60°, and 90°, Figure 5b–d) with a constant bending speed of approximately 10°/s and a 5 s hold at each angle followed by 5 s of relaxation. The relative resistance change (ΔR/R_0_) exhibited a highly synchronized and progressive response, reaching distinct plateaus of approximately −40%, −60%, and −70%, respectively. The cyclic waveforms were highly reproducible without baseline drift, highlighting the device’s stable performance in differentiating precise joint articulations. The use of flexible pressure sensors for finger motion detection and gesture recognition has been widely explored, with recent studies achieving high recognition accuracy through deep learning algorithms [34]. When attached to the knee joint during running (Figure 5g) at a treadmill speed of approximately 8 km/h, the sensor reliably registered intense pressure variations, producing sharp, consistent response peaks with amplitudes nearing −75% across multiple continuous gait cycles. Compared with the uniform porous membrane (UPM), which shows signal saturation above 50 kPa (Figure 3c), the GPM maintains a detectable response up to 120 kPa due to its gradient architecture. This advantage is particularly evident in high-pressure applications such as running. For subtle physiological signal detection, the sensor was affixed to the inner surface of a surgical mask (Figure 5e) and recorded respiration at a normal rate of approximately 12–15 breaths per minute, outputting smooth and periodic resistance fluctuations (ΔR/R_0_ ≈ −1.5%) that perfectly matched the inhale-exhale cycles. Wearable respiratory monitoring systems have been integrated into face masks for continuous health assessment, with some smart masks capable of simultaneous pressure sensing and biochemical analysis [35]. When placed over the thyroid cartilage (Figure 5f), the device captured vocal vibrations during pronunciation of monosyllabic (“hi”), disyllabic (“hello”), and trisyllabic (“banana”) words at normal speaking volume, each repeated three times, generating distinct waveform envelopes: a sharp single peak for a monosyllabic sound, a double-peak pattern for a disyllabic sound, and a triple-peak pattern for a trisyllabic sound. This demonstrates its rapid transient response and potential for non-invasive voice recognition systems. Throat-attachable pressure sensors have recently been combined with deep learning to enable accurate dialect and word recognition by detecting muscle vibrations during vocalization [36]. To further demonstrate spatial pressure sensing capability, a 4 × 4 sensor array was fabricated (Figure 5h). The array was tested under three different pressure distribution patterns. Under random pressure loading (Figure 5i), the corresponding pressure map clearly resolved the positions where pressure was applied. Under uniform pressure loading (Figure 5j), the five sensors in the same row showed similar signal intensity with a coefficient of variation of approximately 12% across the 16 sensing units, indicating good response consistency. Under gradient pressure loading (Figure 5k), the histogram height increased progressively along the vertical direction, demonstrating the array’s ability to quantitatively distinguish pressure levels [37]. For clarity, in Figure 5i–k, the *X* and *Y* axes represent the row and column positions of the 4 × 4 sensor array, respectively, and the histogram height represents the relative resistance change (ΔR/R_0_). These results confirm the sensor’s potential for wearable health monitoring, human–machine interfaces, and electronic skin applications requiring spatial pressure mapping. A comparison of the sensing performance between our GPM sensor and previously reported flexible piezoresistive sensors [29,38,39,40,41,42] is provided in Table S1 (Supporting Information). Our sensor achieves a competitive balance between sensitivity (−3.05 kPa^−1^ in 0–20 kPa) and working range (0–120 kPa), with the advantages of a simple bilayer sugar-templating approach and a water-processable, biocompatible PVA matrix.

## 4. Conclusions

In summary, a gradient porous PVA/CB-based flexible piezoresistive sensor was successfully developed using a simple and eco-friendly sugar-templating method. The bilayer structure, consisting of a small-pore bottom layer and a large-pore top layer, enables a sequential deformation mechanism that effectively balances sensitivity and working range. The sensor exhibits a high sensitivity of −3.05 kPa^−1^ in the low-pressure range (0–20 kPa) and maintains a stable response up to 120 kPa, outperforming uniform porous structures which suffer from early saturation above 50 kPa. In addition, the sensor demonstrates fast response (265 ms), rapid recovery (203 ms), and good cycling stability over 1000 repeated loading–unloading cycles. Practical applications in respiration monitoring, vocal vibration detection, finger bending, and running have been successfully demonstrated, and a 4 × 4 sensor array shows promising capability for spatial pressure mapping. This work provides a low-cost, scalable, and water-processable strategy for developing high-performance flexible pressure sensors with balanced overall performance, offering great potential for wearable health monitoring and human–machine interface applications.

## Data Availability

The original contributions presented in this study are included in the article/Supplementary Material. Further inquiries can be directed to the corresponding author.

## Supplementary Materials

The following supporting information can be downloaded at: https://www.mdpi.com/article/10.3390/polym18131630/s1.
