# Supplementary material for: Gradient Porous PVA/CB Composites for High-Performance Flexible Piezoresistive Sensors
Source: Polymers (Basel). 2026 Jun 30;18(13):1630. doi: 10.3390/polym18131630 (PMC13364449; doi:10.3390/polym18131630)
Supplement: Supplementary file 1 [file polymers-18-01630-s001.zip › polymers-4369466-supplementary.pdf]

Gradient Porous PVA/CB Composites for High-Performance Flexible Piezoresistive Sensors

Changze Mei [a,b], Tian Zhang [a,b] and Yong Zhang [a,b],*

[a]State Key Laboratory of Advanced Glass Materials, School of Materials Science and Engineering, Wuhan University of Technology, Wuhan 430070, China
[b]Center for Smart Materials and Device Integration, Wuhan University of Technology, Wuhan 430070, China
[c]Electronic Information School, Wuhan University, Wuhan 430072, China
[d]Suzhou Institute of Wuhan University, Suzhou 215000, China

*Corresponding author.
E-mail: zhangyong123@whut.edu.cn (Y. Zhang)

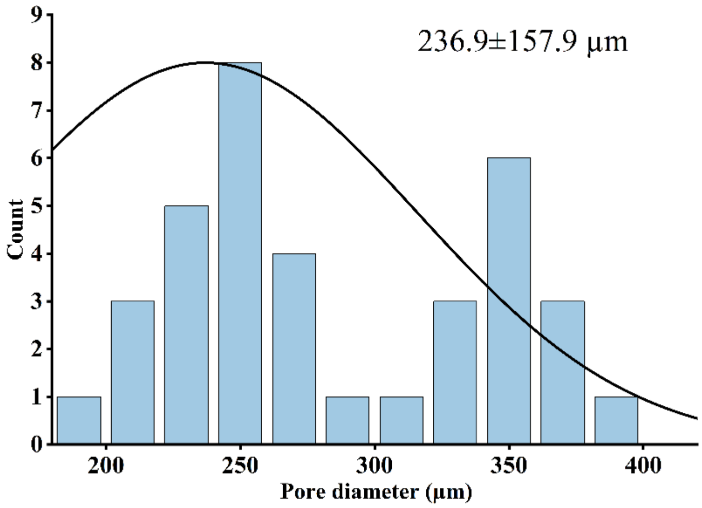

Figure S1. Pore size distribution histogram of the GPM obtained from ImageJ analysis of cross-sectional SEM images. The distribution shows a broad range from approximately 150 µm to 400 µm, reflecting the coexistence of small pores (bottom layer) and large pores (top layer).

Table S1. Comparison of this work with previously reported flexible piezoresistive pressure. sensors

Materials	Sensitivity (kPa^{-1})	Pressure range (kPa)	Respond time /recover time	Stability
MXene/GOQDs/MS aerogel[1]	4.384% kPa^{-1}	1-80 kPa	246/461 ms	2500
$Ti_3C_2T_x$/MWCNTs[2]	-4.58 kPa^{-1}	0-150 kPa	133/253 ms	4000
TPU/MWCNT [3]	69.8 kPa^{-1}	0-300 kPa	5/5 ms	5000
PVC/CNT[4]	5.57 kPa^{-1}	0-120 kPa	80/50 ms	500
Loofah fiber fabric (LFF) / MWCNTs[5]	30.8 kPa^{-1}	0-140 kpa	64/68 ms	5500
PVA gradient ionogel[6]	1.09 kPa^{-1}	0-800 kpa	92 ms	5000
This work	-3.05 kPa^{-1}	0-120 kpa	265/220 ms	1000

1. Wang, J.; Ma, H.; Ma, T.; Zhang, Z.; Wang, G.; Si, F.; Ding, J.; Zhang, W.; Fan, X. A Sensitive Flexible Pressure Sensor Based on MXene/GOQDs/MS Aerogel for Machine Learning Powered Human Sitting Posture Recognition. Microchem. J. **2025**, *218*, 115494. https://doi.org/10.1016/j.microc.2025.115494
2. Zhou, Y.; Han, B.; Ma, P. $Ti_3C_2T_x$/MWCNTs Based Sandwich-Type Hybrid Microstructure for High-Performance Flexible Pressure Sensor. Mater. Lett. **2025**, *399*, 139090. https://doi.org/10.1016/j.matlet.2025.139090
3. Bai, N.; Xu, D.; Su, Z.; Li, G.; He, L.; Chen, Y.; Guo, C.; Zhou, L.; Qin, X.; Zhang, J.; Wu, D.; Wang, W. Dual-Graded Microstructure Engineering for Flexible Piezoresistive Sensors with High Sensitivity and Broad Linear Range in Physiological Monitoring. Adv. Sci. **2025**, *12*, e07135. https://doi.org/10.1002/advs.202507135
4. Zhang, Q.; Liu, Z.; Wu, J.; Sun, P.; Zhang, H. Design and Performance Analysis of a Hybrid Flexible Pressure Sensor with Wide Linearity and High Sensitivity. Sensors **2026**, *26*, 238. https://doi.org/10.3390/s26010238
5. Lu, C.; Shen, Y.; Chan, X.; Yu, S.; Hu, L.; Li, L. Loofah Fiber Fabric-Based Piezoresistive Pressure Sensor with Wide-Linear-Range High Sensitivity. Small **2025**, *21*, 2506521. https://doi.org/10.1002/smll.202506521
6. Zhu, W.; Wang, J.; Sun, W.; Zhou, S.; He, M. Preparation of Gradient Hydrogel for Pressure Sensing by Combining Freezing and Directional Diffusion Processes. Chem. Eng. J. **2023**, *451*, 138335. https://doi.org/10.1016/j.cej.2022.138335
